# Area-to-Length Ratio: a significant predictor of nonunion following intramedullary nailing of comminuted femoral shaft fractures

**DOI:** 10.3389/fsurg.2026.1732494

**Published:** 2026-01-26

**Authors:** Yi Shi, Wei Jiang, Anquan Wang, Xingyi Hua

**Affiliations:** Department of Orthopedics, The First Affiliated Hospital of Anhui Medical University, Hefei, China

**Keywords:** area-to-length ratio, femoral shaft fractures, intramedullary nailing, nonunion, third fragment

## Abstract

**Background:**

The relationship between fragment characteristics and nonunion risk in comminuted femoral shaft fractures remains controversial. This study aimed to investigate the relationship between radiographic fragment features and fracture union outcomes to assist clinical assessment.

**Patients and methods:**

A retrospective analysis was conducted on 91 femoral shaft fractures from 89 patients. Patient demographics, injury characteristics, and radiographic parameters—including fragment size, displacement, gap area, nail-to-canal diameter ratio, and ALR (gap area/major fragment length)—were analyzed. Univariate and multivariate logistic regression were used to identify independent risk factors for nonunion.

**Results:**

The overall nonunion rate was 16.5% (15/91). Univariate analysis revealed that a larger gap area (OR = 1.004, *P* < 0.001) and a lower distal nail-to-canal diameter ratio (OR = 0.013, *P* = 0.023) were associated with nonunion. The novel ALR parameter demonstrated a strong association with nonunion (OR = 2.349, *P* < 0.001). In multivariate analysis, ALR remained an independent predictor (OR = 2.304, *P* < 0.001), while traditional factors like fragment size, displacement, and the nail-to-canal ratio were not significantly associated.

**Conclusion:**

Traditional radiographic measures such as fragment length, width, Nail-to-canal ratio and absolute displacement did not show a significant association with healing outcome in our cohort. While the ALR is a significant predictor of nonunion in comminuted femoral shaft fractures, may assist in the early identification of cases at higher risk for nonunion, which could inform clinical vigilance regarding the potential need for more intensive management strategies.

## Introduction

Femoral shaft fractures are common orthopedic injuries, predominantly resulting from high-energy trauma such as traffic accidents or falls from heights. Intramedullary nailing (IMN) has become the gold standard for treating these fractures due to its advantages in load-sharing stability, high union rates, and minimal soft tissue disruption (1). However, nonunion remains a significant complication, with reported incidences ranging from 5% to 33% in fractures involving third fragments (2). Nonunion not only prolongs patient recovery but also increases socioeconomic burdens due to delayed rehabilitation and potential revision surgeries.

Existing studies have identified several risk factors for nonunion, including fragment displacement, size, and patient-specific variables. For instance, Lee et al. demonstrated that third fragment displacement exceeding 20 mm proximally or 10 mm distally significantly reduced union rates (3). Similarly, Vicenti et al. highlighted that fragments larger than 40 mm in length or displaced by ≥12 mm were independent predictors of delayed healing (4). The degree of displacement has been further stratified, with severe displacement or fragment turnover (Grade III/IV) leading to dramatically lower union rates (13.3%–28.6%) compared to minimal displacement (Grade I, 89.2%) (5). However, other studies on tibial butterfly fragments found that fragment size itself did not differ between union and nonunion groups, whereas combined fragment displacement was significantly higher in nonunions (6), suggesting the critical role of the resulting fracture gap. Conversely, some studies have argued that the mere presence or morphology of the wedge fragment may be secondary to the quality of reduction between the main fragments (7), or that open reduction and fixation of the fragment may even be detrimental to healing by disrupting blood supply (8, 9). Conflicting findings also exist regarding other radiographic factors, such as the nail-to-canal diameter ratio and the length of the unfixed distal segment (10, 11). Despite these findings, controversies persist regarding the relative importance of fragment morphology vs. displacement, as well as the impact of newer parameters such as the “fragment width ratio” proposed by Lin et al. (12).

Current literature primarily focuses on absolute measurements of fragment size or displacement, yet few studies integrate these variables. Furthermore, most evidence derives from small cohorts or single-center analyses, limiting generalizability. For example, Hamahashi et al. emphasized displacement as the dominant risk factor in a retrospective study of 51 patients (13), while Yin et al. argued that fragment circumference and displacement synergistically impair healing (14). Recently, Yoon et al. found the size of the main fracture gap is associated with bone healing, rather than the wedge fragment (7). These discrepancies underscore the complexity of predicting healing and suggest that isolated morphological measurements may be insufficient.

While existing parameters like the width ratio focus on the fragment's own proportions, we developed the Area-to-Length Ratio (ALR) to directly quantify the relationship between the bone defect (gap area) and the potential stabilizing biological unit (major fragment length). This study aims to investigate whether this integrative metric provides superior predictive value for nonunion compared to traditional, isolated morphological measures.

In this study, we retrospectively analyzed 91 cases of femoral shaft fractures treated with IMN to systematically evaluate risk factors for nonunion. By incorporating fragment characteristics (size, displacement, width ratio), patient demographics, and surgical variables, we aim to clarify the interplay of these factors and identify high-risk populations. Our findings may assist in intraoperative decision-making, such as bone grafting or early intervention, to mitigate nonunion risks and improve clinical outcomes.

## Materials and methods

### Patient selection

From October 2017 to December 2024, a total of 102 patients with comminuted femoral shaft fractures treated with intramedullary nailing (IMN) in the First Affiliated Hospital of Anhui Medical University were initially reviewed. The inclusion criteria were as follows: 1. age between 18 and 75 years; 2. acute femoral shaft fracture classified as AO/OTA type 32-B or 32-C; 3. treatment with closed reduction and internal fixation using an intramedullary nail. Exclusion criteria included: 1. pathological fractures; 2. presence of systemic or local infection at the fracture site; 3. previous ipsilateral femoral shaft fracture; 4. loss to follow-up or incomplete clinical/radiological data beyond 10 months postoperatively.

**Figure 1:**
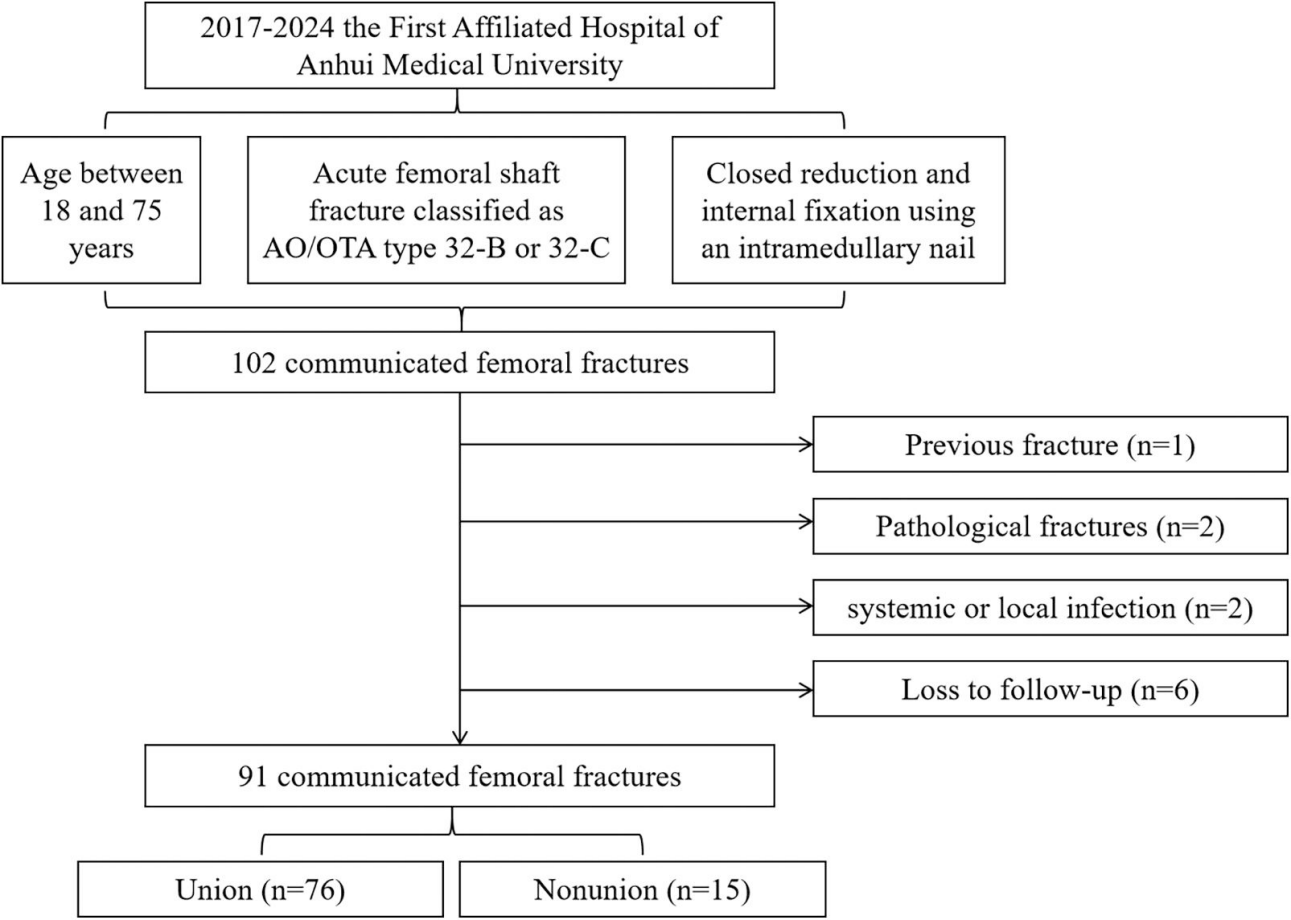
Flow chart for screening patients with femoral shaft fractures following inclusion and exclusion criteria.

A total of 89 patients met the inclusion criteria, including 2 cases of bilateral femoral shaft fractures, resulting in 91 fractures available for analysis (Figure 1). All patients provided informed consent, and the study was approved by the institutional ethics committee.

### Surgical technique and postoperative management

All surgeries were performed by experienced orthopedic surgeons at our institution. Patients underwent IMN within 10 days after injury. Under supine positioning, closed reduction was achieved using a traction table. Sequential reaming was performed, followed by insertion of the intramedullary nail and interlocking screws at both proximal and distal ends.

Postoperatively, patients were encouraged to perform early functional exercises of the affected limb. Standardized protocols for analgesia, infection prophylaxis, and anticoagulation with low-molecular-weight heparin were administered. Partial weight-bearing with crutches was permitted under guidance. Follow-up evaluations were conducted at 3-month intervals for a minimum of 10 months.

### Definition of fracture union and nonunion

Fracture union was defined as: 1. radiographic evidence of callus formation, blurred or disappeared fracture lines, and bony bridging across at least three cortices on x-ray or CT scans within 10 months postoperatively; 2. ability to walk unaided on level ground for at least 3 min (15).

Nonunion was defined as: 1. failure to meet the above criteria by 10 months postoperatively; 2. requirement for secondary interventions such as bone grafting or dynamization to promote healing.

### Data collection and radiographic measurements

Patients were divided into union and nonunion groups. General patient characteristics were recorded, including age, gender, smoking status, diabetes mellitus, open fracture status, and time from injury to surgery.

**Figure 2:**
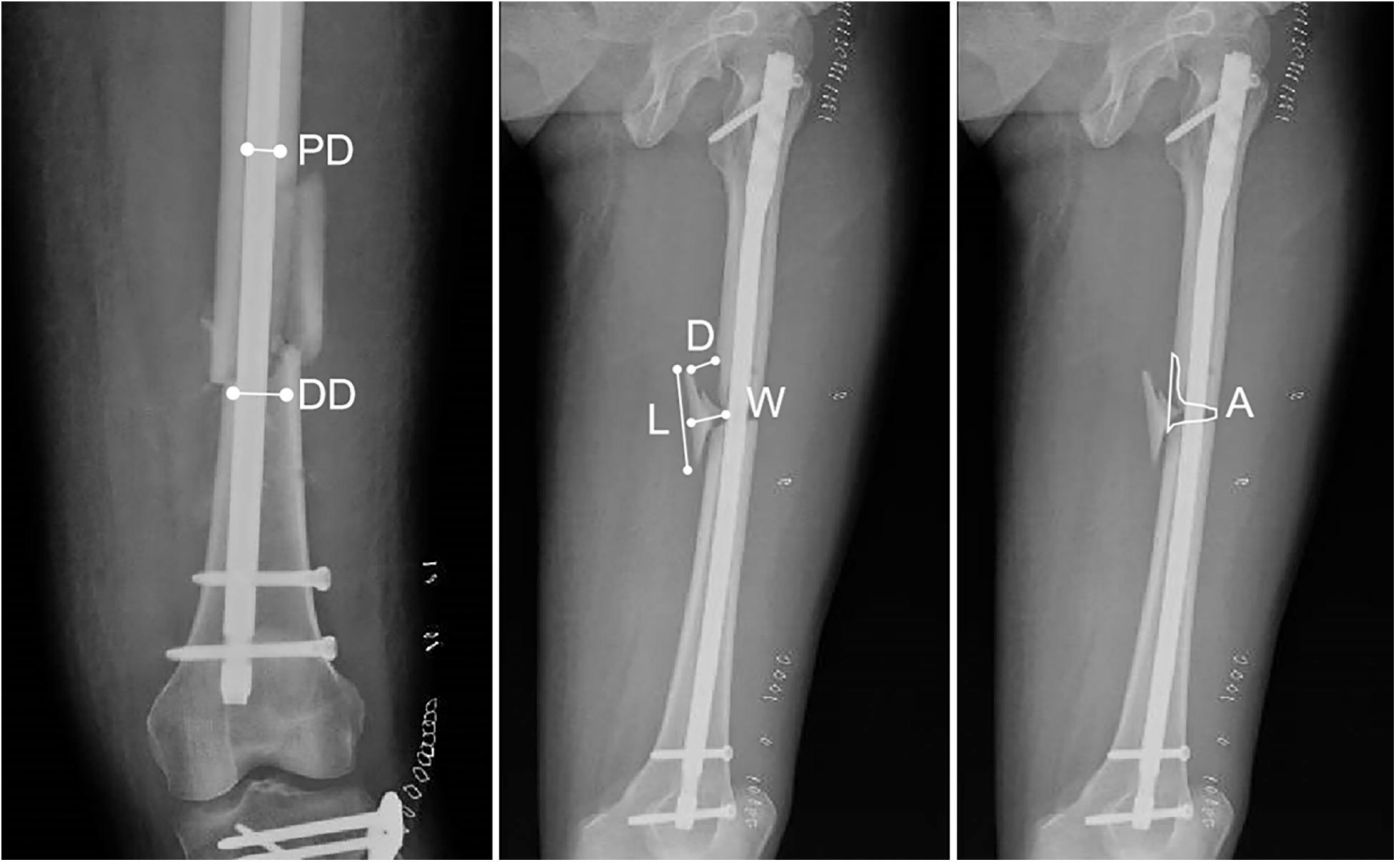
Postoperative radiographic measurements. PD: canal diameter at the proximal end of fracture. DD: canal diameter at the distal end of fracture. D: displacement of fragment. L: length of fragment. W: width of fragment. A: area of the fracture gap.

Radiographic parameters were measured by a single observer and included: Major fragment length: the longest longitudinal dimension of the largest free fragment; Major fragment width: the maximum dimension perpendicular to the fragment length; Fragment displacement: the sum of the maximum distances from the fragment to its anatomical position on anteroposterior and lateral views; Gap area between proximal and distal fragments: the area of bone defect between the main fracture fragments; Nail-to-canal diameter ratio (NCR): measured at both proximal and distal fracture levels; Area-to-length ratio (ALR): calculated as the gap area divided by the major fragment length. These parameters were shown in Figure 2.

### Statistical analysis

Continuous variables were tested for normality using the Shapiro–Wilk test. Normally distributed data were analyzed using independent samples *t*-test, while non-normally distributed data were analyzed with the Mann–Whitney *U* test. Categorical variables were compared using the chi-square test (16).

Univariate logistic regression was performed to identify factors associated with fracture union. Variables with *p* < 0.05 in univariate analysis were included in a multivariate logistic regression model. A *p*-value <0.05 was considered statistically significant. All analyses were performed using SPSS version 26.0.

## Results

### Patient characteristics

Between October 2017 and December 2024, 102 patients with comminuted femoral shaft fractures were initially identified. Of these, 89 patients met the inclusion criteria, including 2 cases of bilateral fractures, resulting in a total of 91 femoral shaft fractures available for analysis.

The cohort had a mean age of 45.74 ± 15.23 years (range: 19–74 years). According to the defined criteria, 76 fractures (83.5%) achieved union, while 15 (16.5%) developed nonunion (Table 1). The union group consisted of 50 males (65.8%), with a mean age of 45.21 ± 14.78 years. The nonunion group included 9 males (60.0%), with a mean age of 48.40 ± 16.83 years. The average time from injury to surgery was 3.29 ± 1.41 days, with no significant difference between the union (3.22 ± 1.29 days) and nonunion groups (3.60 ± 1.77 days).

**Table 1:** Patient demographics and baseline characteristics.

Variables	Total (*n* = 91)	Union group (*n* = 76)	Nonunion group (*n* = 15)	*P* value
Age	45.74 ± 15.235	45.21 ± 14.778	48.40 ± 16.834	0.219
Gender (male)	59 (64.84%)	50 (65.79%)	9 (60%)	0.769
Open fracture	4 (4.40%)	3 (3.95%)	1 (6.67%)	0.52
Diabetes melitus	9 (9.89%)	7 (9.21%)	2 (13.33%)	0.457
Smoking	7 (7.69%)	4 (5.26%)	3 (20%)	0.085
AO type (type C)	14 (15.38%)	12 (15.79%)	2 (12.33%)	0.584
Time from injury to surgery	3.29 ± 1.413	3.22 ± 1.292	3.60 ± 1.765	0.1

**Figure 3:**
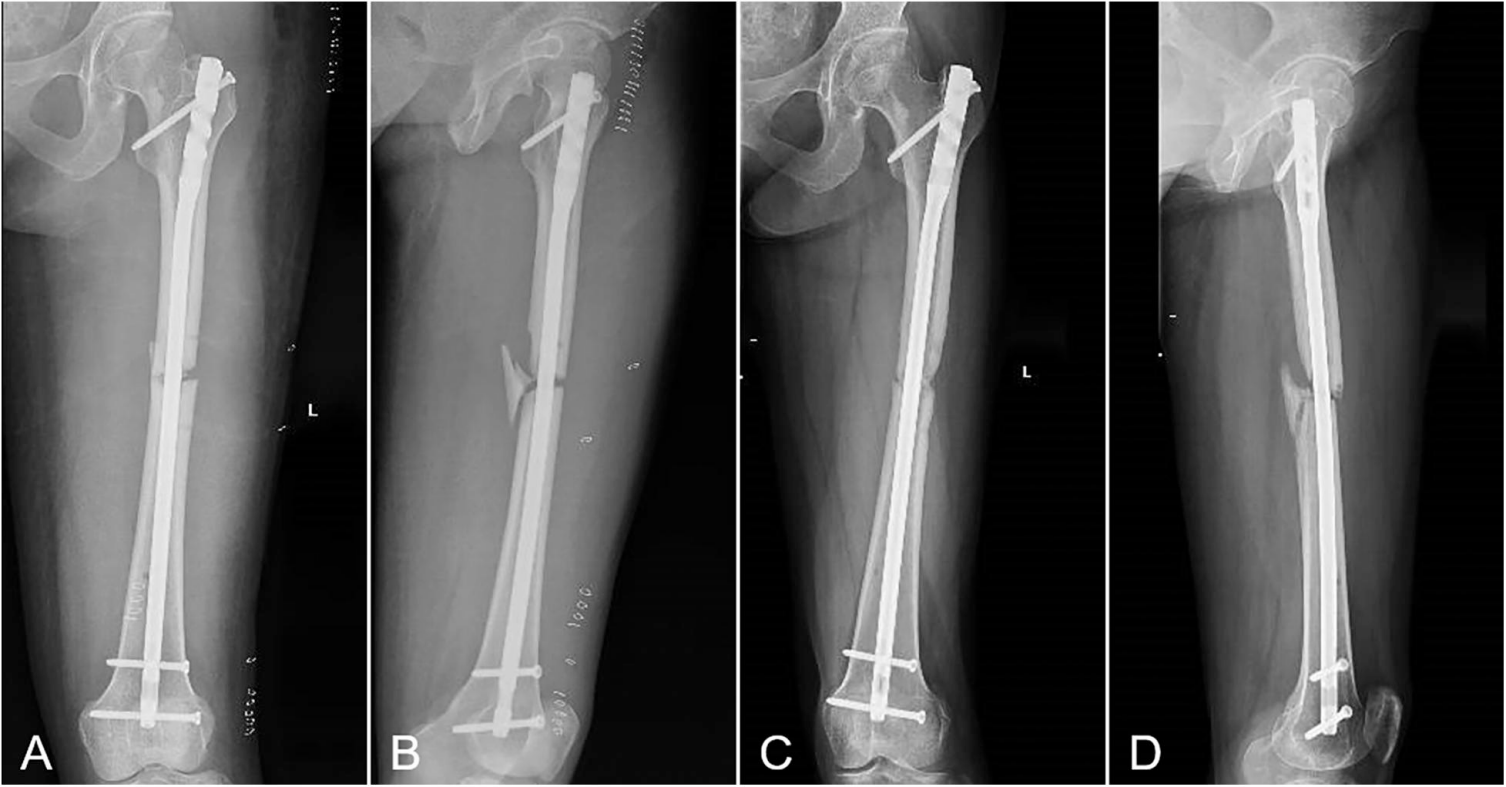
A case of fracture nonunion. **(A,B)** Immediate postoperative anteroposterior **(A)** and lateral **(B)** radiographs of a patient who subsequently developed nonunion. **(C,D)** Follow-up anteroposterior **(C)** and lateral **(D)** radiographs of the patient at 10 months postoperatively, confirming nonunion with no bridging callus across the fracture gap.

**Figure 4:**
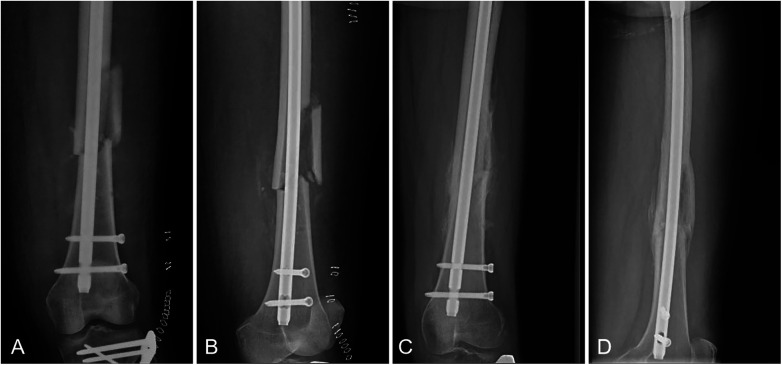
A case of fracture union.**(A,B)** Immediate postoperative anteroposterior **(A)** and lateral **(B)** radiographs of a patient who achieved fracture union. **(C,D)** Follow-up anteroposterior **(C)** and lateral **(D)** radiographs of the same patient at 10 months, demonstrating successful fracture union with robust bridging callus formation.

Among the 91 fractures, 4 were open fractures, of which 3 healed and 1 progressed to nonunion. Nine patients had diabetes mellitus, 2 of whom developed nonunion. Seven patients were smokers, 3 of whom experienced nonunion. According to the AO/OTA classification, 77 fractures were type B and 14 were type C. Among the type C fractures, 12 healed and 2 resulted in nonunion (Figures 3, 4).

Comparative analysis of baseline characteristics between the union and nonunion groups revealed no statistically significant differences in age, sex, time to surgery, open fracture status, diabetes, smoking history, or AO/OTA fracture type (Table 1).

### Univariate analysis of radiographic parameters

All radiographic parameters were continuous variables and were evaluated as potential risk factors using univariate logistic regression (Table 2). As shown in Table 2, no significant associations were found between fracture union and major fragment length (*p* = 0.486), fragment width (*p* = 0.296), degree of fragment displacement (*p* = 0.365), or the NCR at the proximal fracture level (*p* = 0.383).

**Table 2:** Univariate logistic analysis of radiographic parameters.

Variables	*B*	SE	Wald	OR	*P*
Major fragment length	−0.004	0.006	0.486	0.996	0.486
Major fragment width	−0.035	0.034	1.093	0.965	0.296
Fragment displacement	0.02	0.022	0.821	1.02	0.365
Gap area	0.004	0.001	12.346	1.004	<0.001
NCR (proximal)^a^	3.673	4.213	0.760	39.365	0.383
NCR (distal)^b^	−4.368	1.915	5.202	0.013	0.023
ALR	0.854	0.203	17.726	2.349	<0.001

NCR, nail-to-canal diameter ratio.

a NCR at proximal end of the fracture.

b NCR at distal end of the fracture. ALR: Area-to-length ratio, calculated as the gap area divided by the major fragment length.

However, a larger gap area between the main fragments was significantly associated with an increased risk of nonunion (OR = 1.004, *p* < 0.001). A lower NCR at the distal fracture level also correlated with a higher risk of nonunion (OR = 0.013, *p* = 0.023).

Furthermore, a novel parameter—the area-to-length ratio (ALR), defined as the gap area divided by the major fragment length—was introduced and showed a strong association with nonunion (OR = 2.349, *p* < 0.001).

### Multivariate logistic regression analysis

To further assess the independence of the significant variables from univariate analysis, the distal NCR and ALR were included in a multivariate logistic regression model (Table 3). Given the suspected associations of fragment displacement, diabetes, and smoking with fracture union, these variables were included in the multivariate analysis to ensure a comprehensive assessment of potential risk factors. The results indicated that the distal NCR, fragment displacement, diabetes and smoking were not independently associated with fracture union. In contrast, a higher ALR remained a significant predictor of nonunion (OR = 2.304, *p* < 0.001).

**Table 3:** Multivariate logistic regression.

Variables	*B*	SE	Wald	OR	*P*
NCR(distal)	−2.438	3.246	0.564	0.087 (0.001–50.626)	0.453
ALR	1.099	0.292	14.120	3.000 (1.692–5.322)	<0.001
Fragment displacement	−0.097	0.062	2.443	0.907 (0.803–1.025)	0.118
smoking	1.379	1.605	0.738	3.970 (0.171–92.307)	0.390
diabetes	−2.521	2.469	1.042	0.080 (0.001–10.169)	0.307

NCR, nail-to-canal diameter ratio; ALR, area-to-length ratio, calculated as the gap area divided by the major fragment length.

**Figure 5:**
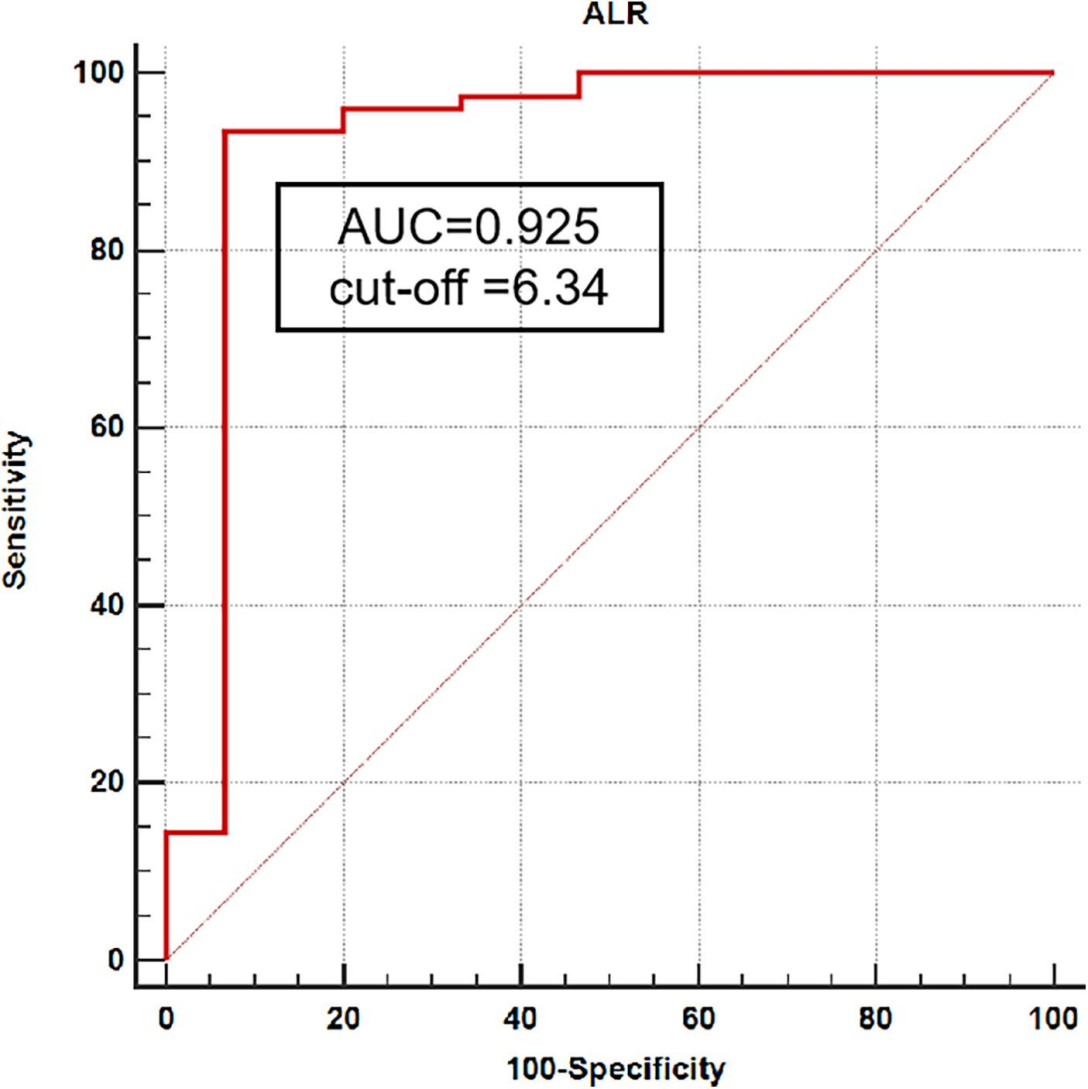
A receiver operating characteristic curve (ROC) was generated using the ALR as a predictor of nonunion to demonstrate a threshold value. Cut-off = 6.34 has a sensitivity of 0.93 and a specificity of 0.93 in predicting the occurrence of nonunion.

The discriminatory capacity of ALR for predicting nonunion was quantified using receiver operating characteristic (ROC) curve analysis. The area under the curve (AUC) was 0.925 (95% CI: 0.873–0.977), indicating excellent discriminative ability. The optimal cutoff value for ALR was determined to be 6.34, which yielded a sensitivity of 0.93 and a specificity of 0.93 for identifying nonunion (Figure 5).

These findings underscore the clinical importance of achieving adequate reduction of the fracture gap during surgery, particularly in cases involving shorter intermediate fragments that need to be adequately reduced between the main fracture fragments.

## Discussion

The principal finding of this study is that the Area-to-Length Ratio (ALR)—a novel composite parameter combining the gap area between the main fracture fragments and the length of the major third fragment—is an independent predictor of nonunion in femoral shaft fractures treated with IMN. In contrast, traditional radiographic measures such as fragment length, width, NCR and absolute displacement did not show a significant association with healing outcome in our cohort.

The strong correlation between a higher ALR and increased risk of nonunion can be explained from both biological and biomechanical perspectives. A large gap area indicates substantial bone loss at the fracture site, which exceeds the critical-sized defect capable of spontaneous bridging. Concurrently, a smaller third fragment (reflected by a lower denominator in the ALR calculation) possesses limited osteogenic potential and provides inadequate structural support to sustain mechanical stability under axial and torsional loads. This combination creates an unfavorable environment for bone healing, characterized by insufficient biological stimulus and excessive interfragmentary strain, ultimately leading to failed union. This aligns with the “critical-size defect” concept discussed by Lin et al., who emphasized the importance of relative fragment dimensions in healing prognosis (12, 17).

Our findings are further supported by a recent study by Yoon et al. (7), which specifically investigated the role of wedge fragments in femoral shaft fractures treated with IMN. In their cohort of 95 patients, they found that wedge fragment characteristics—including size, displacement, angle, and reversal morphology—did not significantly influence union rates. Instead, the size of the main fracture gap was the only radiographic factor significantly associated with nonunion (5.2 mm in the union group vs. 15.6 mm in the nonunion group, *p* = 0.01). Layon et al. indicated that even flipped intercalary fragments can achieve a union rate as high as 92% following closed reduction and intramedullary nailing (18). This reinforces the notion that the primary determinant of healing is not the morphology of the intermediate fragment *per se*, but the integrity of the main fracture environment and the adequacy of reduction between the principal proximal and distal segments.

Our results both corroborate and refine the existing literature. The study by Lin et al. demonstrated that fragment displacement >10 mm significantly impaired fracture healing, with union rates of 75.9% in the small-gap (≤10 mm) group vs. only 21.1% in the large-gap (>10 mm) group (19). They further highlighted that inverted fragment morphology represented the most unfavorable condition for union. Louka et al. found that displacement ≥14 mm increased the risk of delayed healing or nonunion by approximately 15-fold (20). Our findings are consistent with their emphasis on displacement as a critical factor, while our ALR parameter provides a more comprehensive assessment by simultaneously considering both the defect size (gap area) and the potential stabilizing effect of the fragment (length). This integrated approach may explain why ALR demonstrated stronger predictive value than displacement alone in our multivariate model. Furthermore, while Lee et al. and Vicenti et al. identified absolute fragment displacement (≥20 mm) and size (≥8 cm) as risk factors (3, 4), our data suggest that the relative relationship between the fracture gap and the fragment size may be more critical. A large fragment may still facilitate healing if it effectively bridges a small gap, whereas a small fragment may be insufficient to stabilize a large defect. This could explain why some studies reported conflicting results regarding the influence of fragment size alone.

The significance of the fracture site environment in predicting nonunion is further underscored by a recent study focusing on the most severe pattern of femoral shaft fractures. Cho et al. identified the “exposed nail length”—a parameter reflecting the extent of cortical defect and lack of bony containment—as a sole independent risk factor for nonunion, with a cut-off value of 19.1 mm (21). This finding resonates with our results in several key aspects. Firstly, both studies suggest that traditional fragment characteristics like size and displacement may be insufficient predictors, especially in complex fracture patterns. Secondly, both our ALR and the “exposed nail length” conceptually measure a “critical defect” at the fracture site, whether it is a volumetric gap relative to fragment size (ALR) or a segmental lack of cortical support for the implant. While the ALR is potentially applicable to a broader range of comminuted fractures (32-B/C) and can be assessed preoperatively, the “exposed nail length” provides a crucial post-operative warning sign in the most severe cases. Together, these findings highlight a paradigm shift towards evaluating the integrity of the fracture environment and the magnitude of the “bone defect” rather than relying solely on the morphology of individual fragments.

Interestingly, the NCR at the distal level showed significance in univariate analysis but lost its independent predictive value in the multivariate model when ALR was included. This indicates that the quality of fracture reduction and the morphology of the fracture microenvironment, as captured by ALR, may outweigh the influence of implant fit in the distal segment on the ultimate healing outcome.

The predictive value of ALR has direct implications for the ongoing debate on how to manage the third fragment. Some evidence suggests that aggressive open reduction and fixation may compromise vascularity and lead to worse outcomes (8). Gambuti et al. similarly concluded that open reduction of the third fragment was a significant predictor of complications, whereas fragment characteristics themselves were not (22). In this context, a preoperative or intraoperative assessment yielding a high ALR could be pivotal. It may identify cases where the inherent risk is so high that achieving an excellent closed reduction is paramount, or where early consideration of biological augmentation (e.g., bone grafting) might be justified, potentially avoiding the pitfalls of fragment manipulation while addressing the root cause of instability.

Several limitations of this study should be acknowledged. First, its retrospective design introduces inherent potential for selection and information bias. Second, the sample size, particularly the number of nonunion cases (*n* = 15), limits the statistical power for extensive subgroup analyses and demands cautious interpretation of the results. A larger, prospective multicenter validation is warranted. Finally, we did not account for certain potentially influential biological factors, such as bone mineral density and uses of drug which could have confounded the healing process.

## Conclusion

In conclusion, the Area-to-Length Ratio (ALR) emerges as a significant and practical radiographic predictor for nonunion in comminuted femoral shaft fractures managed with IMN. It integrates the concepts of bone defect volume and the stabilizing potential of the intermediate fragment into a single quantifiable index. Preoperative recognition of a high ALR may help identify patients at increased risk for nonunion, who might therefore be candidates for closer monitoring or considered for adjunctive procedures such as limited open reduction or bone grafting.

## Data Availability

The original contributions presented in the study are included in the article/Supplementary Material, further inquiries can be directed to the corresponding author.
